# Fermented *Mentha arvensis* administration provides neuroprotection against transient global cerebral ischemia in gerbils and SH-SY5Y cells via downregulation of the MAPK signaling pathway

**DOI:** 10.1186/s12906-022-03653-7

**Published:** 2022-06-25

**Authors:** Md Sadikul Islam, Ha-Young Shin, Yeo-Jin Yoo, Eui-Yong Lee, Ryunhee Kim, Young-Jin Jang, Md Rashedunnabi Akanda, Hyun-Jin Tae, In-Shik Kim, Dongchoon Ahn, Byung-Yong Park

**Affiliations:** 1 Department of Veterinary Medicine and Biosafety Research Institute, Jeonbuk National University, 79 Gobong-to, Iksan, Jeollabuk-do Republic of Korea 54596; 2 Department of Pharmacology and Toxicology, Faculty of Veterinary, Animal and Biomedical Sciences, Sylhet Agricultural University, Sylhet, 3100 Bangladesh

**Keywords:** Fermented Mentha arvensis (FMA), Ischemic stroke, Neuroprotection, Antioxidant, MAPK

## Abstract

**Background:**

Globally, ischemic stroke is a major health threat to humans that causes lifelong disability and death. *Mentha arvensis* (MA) has been used in traditional medicine to alleviate oxidative stress and inflammation-related disorders. In the present study, the neuroprotective properties of fermented MA (FMA) extract were investigated in the gerbil and SH-SY5Y cells. model of transient global cerebral ischemia.

**Methods:**

Bilateral common carotid artery occlusion-induced transient global cerebral ischemia in gerbil and hydrogen peroxide (H_2_O_2_)-mediated neurotoxic effects in human neuroblastoma cells (SH-SY5Y) were investigated. FMA (400 mg/kg) was orally administered for 7 days before induction of ischemic stroke. To evaluate the neuroprotective activity of FMA, we implemented various assays such as cell viability assay (MTT), lactate dehydrogenase (LDH) assay, histopathology, immunohistochemistry (IHC), histofluorescence, and western blot.

**Results:**

FMA pretreatment effectively decreased transient ischemia (TI) induced neuronal cell death as well as activation of microglia and astrocytes in the hippocampal region. The protective effects of FMA extract against H_2_O_2_-induced cytotoxicity of SH-SY5Y cells were observed by MTT and LDH assay. However, FMA pretreatment significantly increased the expression of the antioxidant marker proteins such as superoxide dismutase-1 (SOD-1) and superoxide dismutase-2 (SOD-2) in the hippocampus and SH-SY5Y cells. Furthermore, the activation of mitogen-activated protein kinase (MAPK) further activated a cascade of outcomes such as neuroinflammation and apoptosis. FMA pretreatment notably decreased TI and H_2_O_2_ induced activation of MAPK (c-Jun N-terminal kinase (JNK), extracellular signal-regulated protein kinase (ERK), and p38) proteins in hippocampus and SH-SY5Y cells respectively. Besides, pretreatment with FMA markedly reduced H_2_O_2_ mediated Bax/Bcl2 expression in SH-SY5Y cells.

**Conclusion:**

Thus, these results demonstrated that neuroprotective activities of FMA might contribute to regulating the MAPK signaling pathway.

**Supplementary Information:**

The online version contains supplementary material available at 10.1186/s12906-022-03653-7.

## Introduction

Stroke is currently the most prevalent cerebral vascular disease and the main reason for permanent disability and mortality [1]. Ischemic and hemorrhagic strokes are the two most common stroke types. When compared to hemorrhagic strokes, the majority of strokes (70–87%) are ischemic and have different causes and mechanisms [2, 3]. In transient global cerebral ischemic stroke, sudden cerebral blood flow is occluded, causing a lack of oxygen and glucose supply to brain tissues leading to irreparable neuronal injury [4]. Transient global cerebral ischemic stroke is a life-threatening cerebrovascular disease in which neuroinflammation and oxidative stress are prominent features [5].

Increased generation of reactive oxygen species (ROS) in neuronal cells during ischemic stroke depletes the antioxidant system causing disruption in the equilibrium between ROS production and consumption. An overabundance of ROS also causes lipid peroxidation with the oxidation of proteins, DNA, and RNA, resulting in neuronal dysfunction and death after cerebral ischemia [6, 7]. Furthermore, hydrogen peroxide (H_2_O_2_) is a common oxidation product and excessive generation has a toxic influence on nerve cell function through Fenton’s reaction, eventually leading to neuronal cell apoptosis [8, 9]. Superoxide dismutases (SODs) are a family of metalloenzymes that induce the conversion of superoxide radicals (O_2_^−^) to H_2_O_2_ and oxygen. SODs are thought to perform a significant role in the treatment of oxidative stress-relevant illnesses as the first line of defense against ROS-mediated damage [10].

In previous studies, mitogen-activated protein kinase (MAPK) was shown to be a crucial mechanism that regulates inflammation and the production of ROS [11]. The three most important kinases involved in the MAPK cascade include extracellular signal-regulated protein kinases (ERK1 and ERK2), c-Jun N-terminal kinase/stress-activated protein kinase (JNK/SAPK), and p38. The phosphorylation of JNK and p38 primarily initiates apoptosis in response to oxidative stress and DNA damage [12]. Similarly, ERK activation mediates apoptosis by induction of mitochondrial cytochrome c release [13]. The Bcl-2 family members provide the most important mechanism for controlling apoptosis and a major site of their action is the mitochondria. The relationship between the pro-apoptotic protein Bax, and the anti-apoptotic protein Bcl-2, regulates mitochondrial-dependent apoptotic cell death by activating the downstream transcription protein caspase-3 [14].

*Mentha arvensis* (MA), also called corn mint, field mint, or wild mint, is a flowering plant in the mint family *Lamiaceae*. The primary active components in MA such as rosmarinic acid, hesperidin, acacetin, diosmin, didymin, linarin, and buddleoside, have been identified in past analyses using high-performance liquid chromatography (HPLC) [15, 16]. The entire plant has several therapeutic properties, including, antioxidant, anti-inflammatory, gastroprotective anti-fungal, and anti-bacterial effects [17–19]. In a previous study, fermentation of MA by *Lactobacillus rhamnosus, Enterococcus faecium,* and *L. acidophilus* increased the active principle of rosmarinic acid and showed stress-induced strong antioxidative effects [19]. In the present study, the neuroprotective effects and underlying mechanism of fermented MA (FMA) were investigated in SH-SY5Y cells and in the gerbil transient global ischemic stroke model.

## Materials and methods

### Preparation of MA var. extract

In this study, the same extract of FMA var. utilized in our previous study was used [19]. In brief, MA was purchased from Omniherb Co. (Daegu, Korea). MA was dried and crushed using a freeze dryer (FDA5508, ilShinbiobase. Co., Ltd., Dongducheon, Korea). For MA fermentation, several bacterial strains including *L. rhamnosus* (L3 KCTC18485P)*, Enterococcus faecium* (L54 KCTC18486P)*,* and *L. acidophilus* (L120 KCTC18487P) were isolated from the feces of mature personal. MA extract was fermented with a 5% mixture of L3, L54, and L120 strains (1:1:1, 1.0 × 10^6^ CFU/mL) at 37 °C for 48 h. Then, the extract was centrifuged (10,000 rpm, 5 min, 4 °C) and the collected supernatant was sterilized at 121 °C for 15 min. The dried extracts were preserved at –20 °C.

### Animals

Six-month-old (bodyweight 65–75 g) male Mongolian gerbils (*n* = 21) were reared according to the animal welfare regulations of the Institutional Animal Care and Use Committee (approval no. CBNU-2020–003) of the Jeonbuk National University Laboratory Animal Center in South Korea. Gerbils were placed in cages and provided sufficient food and fresh water. One week before starting the experiment, gerbils were placed in the laboratory environment for adaptation. During the experiment, animals were housed at 23 ± 2 °C room temperature with 35–60% humidity and a strictly maintained rotation of 12-h dark/12-h light cycles.

### Experimental groups, induction of transient ischemia (TI), and FMA treatment

The gerbils (*n* = 21) were categorized into the following 3 subgroups (*n* = 7 in each group): (1) sham + saline group: subjected to sham surgery and orally treated with saline; (2) transient ischemia (TI) + saline group: subjected to 5 min TI and orally treated with saline; (3) TI + FMA group: subjected to 5 min TI and orally pretreated with FMA extract (400 mg/kg) for 7 days.

For induction of TI, a previously described established protocol was followed [20]. In brief, a 68%, 32%, and 2.5% mixture of nitrous oxide, oxygen, and isoflurane, respectively, was used to anesthetize the gerbils. The common carotid arteries on both sides were separated, and freed of nerve fibers by making a midline ventral incision in the neck and applying non-traumatic aneurysm clips (Yasargil FE 723 K, Aesculap, Tuttlingen, Germany) to occlude both common carotid arteries. The aneurysm-inducing clips were removed after 5 min of blocking. TI-induced pyramidal neuronal death in the hippocampal CA1 area starts after 4 days [21]; therefore, gerbils in all 3 groups were allowed a restoration period of 5 days after induction of TI.

### Histological tissue preparation

According to a previously described protocol [20], after 5 days, all gerbils were intraperitoneally injected with 30% urethane to induce euthanasia and transcardial perfusion was performed with 0.1 M phosphate-buffered saline (PBS, pH 7.4) followed by 4% paraformaldehyde in 0.1 M phosphate buffer (PB, pH 7.4). Then, all brains were carefully collected without any physical damage. The whole-brain tissues comprising the hippocampus were sliced into Sects. 30 µm in diameter using a cryostat (CM1900 UV, Leica, Wetzlar, Germany) and placed into storing solution (Ethylene glycol 30%, 2PO4 10%, distilled water 30%, glycerol 30%) for further analyses.

### Cresyl violet (CV) staining

Cresyl violet (CV) staining was performed to determine the number of live neuronal cells in the gerbil hippocampus. The brain tissue sections containing the hippocampus were placed on microscopy slides coated with gelatin. After 15 min of staining with CV acetate solution (Sigma‐Aldrich, St. Louis, MO, USA), the slides were rinsed in serial ethanol (70%, 80%, 90%, 95%, 100%) baths for dehydration and Canada balsam (Kanto Chemical, Tokyo, Japan) was used to mount the slides.

### Fluoro-Jade B (F-J B) histofluorescence staining

To observe neuronal degeneration in the CA1 area of the hippocampus 5 days after induction of TI, Fluoro-Jade B (F-J B) staining was performed as previously described [20]. In brief, the slides containing tissue sections were immersed in a 1% sodium hydroxide solution. Next, the slides were placed into potassium permanganate (0.06%) solution and then into 0.0004% F-J B (Histochem, Jefferson, AR, USA). The hippocampal tissue sections were observed under a fluorescent microscope (Carl Zeiss, Göttingen, Germany) using a blue (450–490 nm) excitation light and a filter barrier.

### Immunohistochemistry

To examine neuronal damage, the number of activated astrocytes and microglia in the CA1 region after induction of cerebral ischemia, immunohistochemistry was performed following an established technique [20]. In brief, the sections were immersed in 0.3% H_2_O_2_ for quenching. Then, the tissue sections were blocked with 5% goat serum and horse serum. Next, tissue sections were incubated with diluted mouse anti‐NeuN (1:800, Merck Millipore, MA, USA #cat MAB377), rabbit anti-GFAP (1:1,000, GeneTex, Irvine, CA, USA #cat GTX108711), and rabbit anti-Iba-1 (1:1,000, GeneTex #cat GTX 100,042). After incubation, tissue sections were treated with the appropriate secondary antibodies anti-rabbit IgG(Vector Laboratories Inc., Burlingame, CA, USA, #cat BA 1000), anti-mouse IgG (Vector Laboratories Inc., Burlingame, CA, USA, #cat BA 2000) and detected with Vectastain ABC (Vector Laboratories Inc.). Next, diaminobenzidine chromogen in 0.1 M tris–HCL buffer was added to immune-reacted sections for a few seconds. Finally, the tissue sections were dehydrated with graded alcohol and mounted with Canada balsam (Kanto Chemical).

### Histopathological data analysis

Histopathological, histofluorescence and immunohistochemical data were analyzed following the previous method [22]. In short, a digital camera (Axiocam, Carl Zeiss, Germany) and a PC-connected AxioM1 light microscope (Carl Zeiss, Göttingen, Germany) were used to take the images of the hippocampus. The cells of the hippocampal CA1 area were counted in a 250 × 250 μm square using an ImageJ threshold analysis software version 1.52a (NIH, USA). The analyzed tissue sections were chosen at the intervals of 150 μm, and cell counts were calculated by averaging the total cell numbers of six sections collected from each group. Finally, data were converted in the percent (%) and measured in the statistical analysis.

### Cell culture and cell viability analysis

SH-SY5Y (human neuroblastoma cells) cell line was obtained from the *Korean Cell Line* Bank. An equal volume mixture of EMEM (ATCC, Manassas, VA, United States) and Gibco F12 medium (Thermo Fisher Scientific, Waltham, MA, USA) was used to culture the cells. Cell viability was detected using 3-(4,5-dimethylthiazol-2-yl)-2,5-diphenyltetrazolium bromide (MTT; Sigma-Aldrich) [23]. SH-SY5Y cells were pretreated with various dosages of FMA plant extracts (1, 5, 25, or 100 µg/mL) for 2 h and then co-incubated with a freshly prepared 300 µM H_2_O_2_ from a 30% (mass/mass) stock solution for another 24 h. Then, 0.5 mg/mL diluted concentration of MTT solution was added to each well and incubated for 2 h. The formed blue formazan crystals were solubilized with DMSO. Finally, a tunable versa max microplate reader (Molecular Devices, San Jose, CA, USA) was used to measure the optical density at 570 nm absorbance.

### LDH detection assay

Cytotoxicity mediated by H_2_O_2_ was measured by LDH release into the cell culture medium. According to the manufacturer’s instructions, the release of LDH was detected using an LDH cytotoxicity detection kit (Takara, Shiga, Japan). SH-SY5Y cells were pretreated with FMA extract (1, 5, 25 µg/mL) for 2 h and then co-incubated with H_2_O_2_. After 24 h culture medium was aspirated and relative levels of LDH were evaluated by determining the absorbance at 490 nm with a tunable versa max microplate reader (Molecular Devices, San Jose, CA, USA).

### Western blot analysis

RIPA cell lysis buffer (Biosesang, Gyeonggi-do, South Korea) and tissue protein extraction reagent (T-PER, Thermo Scientific, Waltham, MA, USA) were used for protein extraction from the harvested SH-SY5Y cells and Hippocampus of gerbils respectively. The same volume of proteins from each group was separated based on molecular mass using 10–12% SDS‐PAGE. Then, proteins were transferred to nitrocellulose membranes and blocked with 5% bovine serum albumin (BSA; Sigma-Aldrich). Primary antibodies include Beta-actin (Rabbit Cell Signaling, USA, #cat 4970), SOD-1 (Rabbit, Abcam, USA, #cat ab13498), SOD-2 (Rabbit, Abcam, USA, #cat ab 13,533) p-JNK (Rabbit, Cell Signaling, USA, #cat 9251), JNK (Rabbit, Cell Signaling, USA, #cat 9252), p-ERK Rabbit (Rabbit, Cell Signaling, USA, #cat 9101), ERK (Rabbit, Cell Signaling, USA, #cat 9102), p-p38 (Rabbit, Cell Signaling, USA, #cat 9211), p38 (Rabbit, Cell Signaling, USA, #cat 9212), Bcl-2 (Rabbit, Cell Signaling, USA, #cat 3498), Bax (Rabbit Santa Cruz Biotechnology, USA #cat sc-493) were diluted following company guidelines and incubated overnight at 4 °C. Then, the membranes were treated with a secondary antibody (goat anti-rabbit IgG-HRP; Santa Cruz Biotechnology, Inc. Dallas, TX, USA#cat sc2004) for 2 h. Protein expression bands and markers were identified using a clarity western substrate ECL kit (Bio-Rad Laboratories, Hercules, CA, USA). Images were captured using a LAS‐500 image system (GE Healthcare, Little Chalfont, UK). (Blots were cut prior to hybridization with antibodies and displayed the cropped blots in the main paper to improve the clarity and conciseness of the presentation).

### Statistical analysis

GraphPad Prism version 5.0 (GraphPad Software, Inc., La Jolla, CA, USA) was used to calculate and compare all variables among the groups and presented as mean ± standard error of the mean (SEM). Statistical analyses were performed using analysis of variance (ANOVA) followed by Tukey post-hoc tests. A *P-*value < 0.05 for each experiment was considered statistically significant.

## Results

### Evaluation of active compound and content in FMA

In the present study, we used the same FMA extract that was used in our previous experiment to investigate the antistress effect in immobilized rats [19]. Rosmarinic acid (active molecule) was recognized in FMA. The rosmarinic acid content in FMA was 6.32 ± 0.08 mg/g [19].

### Effects of FMA against neuronal cell death induced by TI

**Fig. 1:**
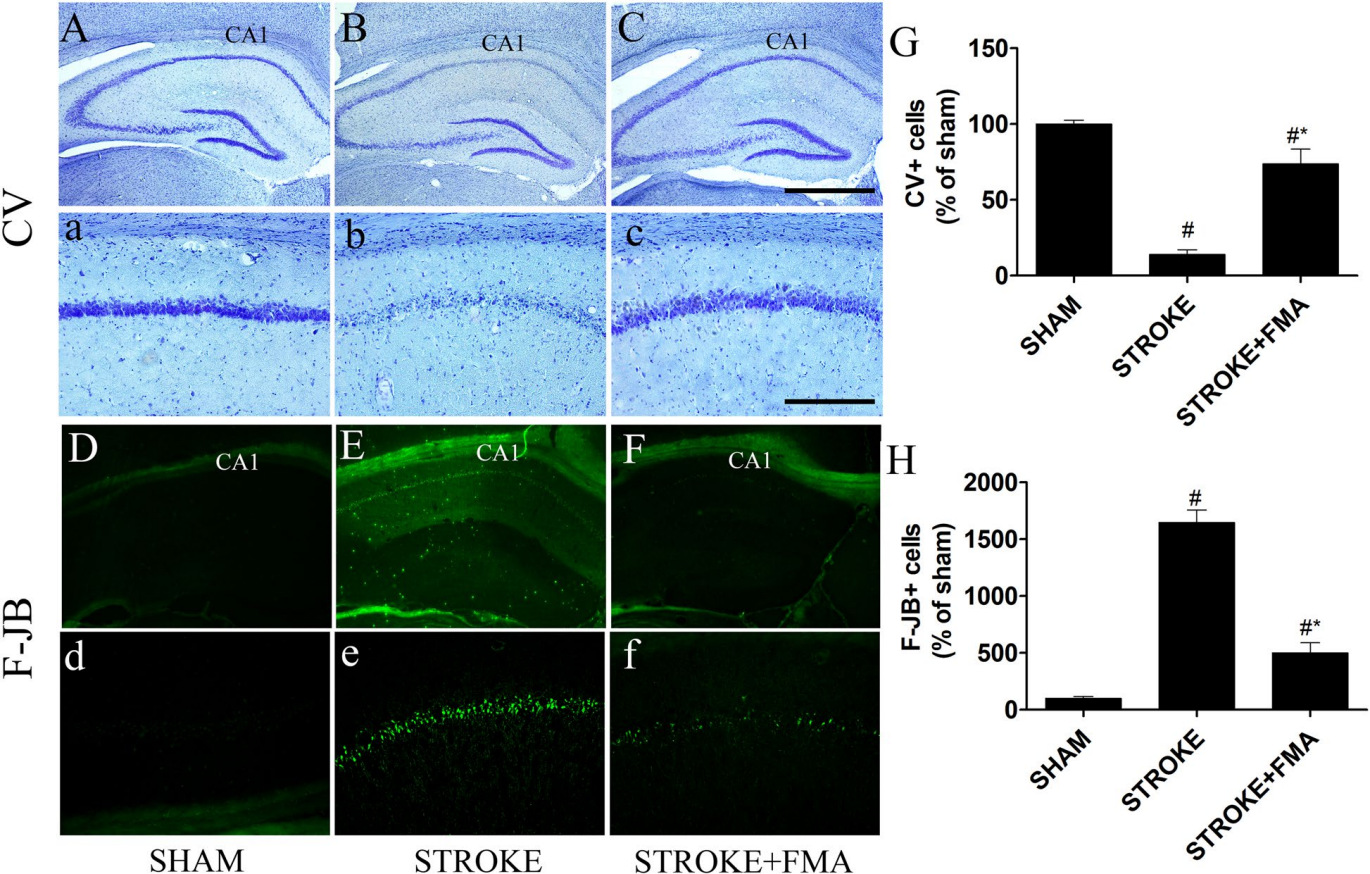
Effects of FMA on CV + cells and F-J B + cells in the hippocampal CA1 area. CV + cells in (**A**) sham group 10x (**a**) 200x, (**B**) TI-induced group 10x, (**b**) 200x, and (**C**) TI-induced + FMA (400 mg/kg) group 10x, (**c**) 200x. F-J B histofluorescence staining of the CA1 area in the (**D**) sham group 10x, (**d**) 200x, (**E**) TI-induced group 10x, (**e**) 200x, and (**F**) TI-induced + FMA (400 mg/kg) group 10x (**f**) 200x. In the TI-induced group, CV + cells were significantly decreased in the CA1 region and an increased number of F-J B + cells were detected in the CA1 region of the hippocampus compared with sham and FMA-pretreated group. (**G)** Graph represent the CV + and (H) F-J B + cells number (% of sham). Data are presented as mean ± SEM, *n* = 3/group, one-way ANOVA with a Tukey post-hoc tests; (#*p* < .05) compared with sham and (*p < .05) compared with Stroke

CV staining was used to determine whether the FMA extract protected against neuronal damage in the CA1 area of the hippocampus after induction of TI (Fig. 1). Large, pyramidal-shaped CV + neuronal cells were easily detected throughout the hippocampus in the sham group (Fig. 1A,a). In the TI-induced group, CV + neuronal cells were markedly decreased in the CA1 area of hippocampus 5 days after TI induction (Fig. 1B,b) compared with the sham group. CV + cells in the TI-induced group were also obviously shrunken with dark nuclei in the CA1 area (Fig. 1 B,b). The cell survival pattern in the CA1 area of the hippocampus in the TI-induced group pretreated with FMA showed a very similar type of CV + neuronal cell arrangement in the sham group (Fig. 1C,c).

TI-induced neuronal degeneration and neuroprotective effects of FMA extract were also determined using F-J B staining (Fig. 1). In the sham group, F-J B + neurons were not observed in the CA1 area of the hippocampus (Fig. 1D,d). However, the CA1 area in the TI-induced group showed a significantly increased number of F-J B + neuronal cells 5 days after TI induction (Fig. 1E,e); pretreatment with FMA extract for 7 days markedly decreased the number of F-J B + neuronal cells in the CA1 region of the hippocampus (Fig. 1F,f).

**Fig. 2:**
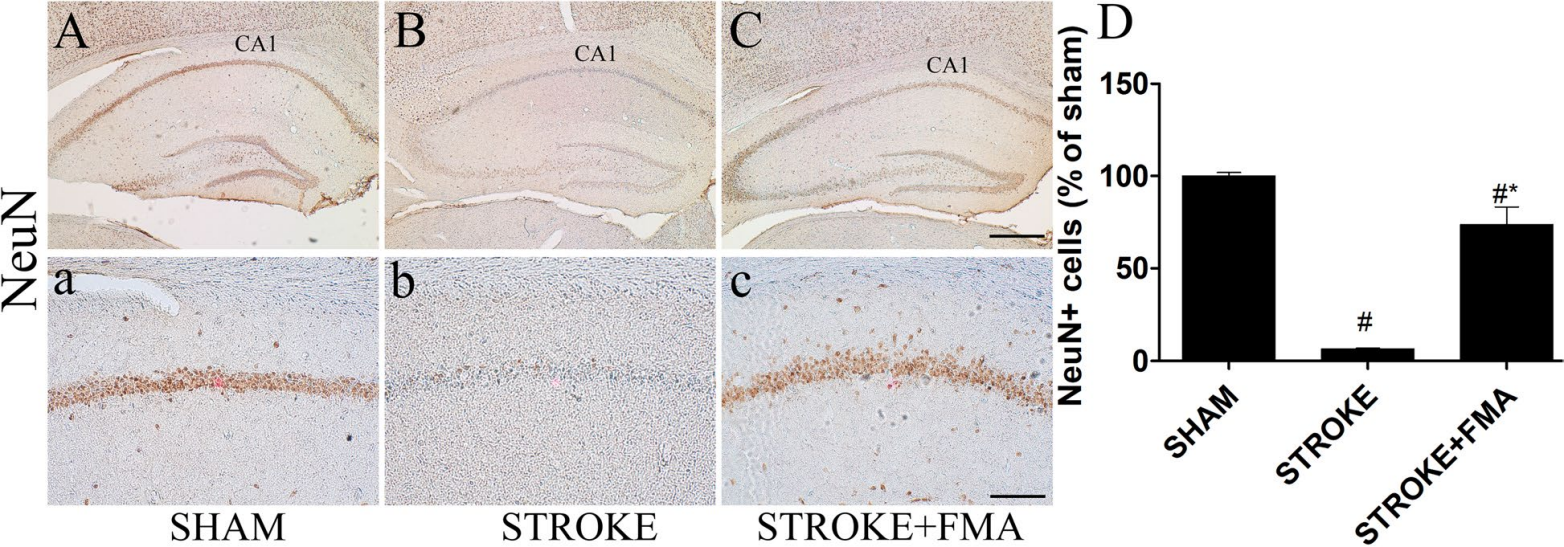
Effects of FMA on NeuN + cells in the CA1 area. NeuN immunoreactive cells in the (**A**) sham group 10x (**a**) 200x, (**B**) TI-induced group 10x, (**b**) 200x, and (**C**) TI-induced + FMA (400 mg/kg) group 10x, and (**c**) 200x. A minimal number of NeuN + neurons was visible in the TI-induced group compared with the FMA-pretreated group. (**D)** Graph represent the immunoreactive NeuN + cells number (% of sham). Data are presented as mean ± SEM, *n* = 3/group, one-way ANOVA with a Tukey post-hoc tests; (#*p* < .05) compared with sham and (*p < .05) compared with Stroke

The protective effects of FMA extract against TI-induced loss of the functional state of neurons in the CA1 area of the hippocampus were identified using NeuN immunohistochemistry (Fig. 2). In the sham group, pyramidal-shaped neurons in the CA1 area were strongly bound with NeuN primary antibody and plainly visible (Fig. 2A,a). However, in the TI-induced group, a markedly decreased number of NeuN + neuronal cells in the CA1 area were detected 5 days after TI induction (Fig. 2B,b). In the FMA-pretreated group, many NeuN + neuronal cells were observed in the CA1 area compared with the TI-induced group (Fig. 2C,c).

### Effects of FMA on TI-induced neuroglia cell activation

**Fig. 3:**
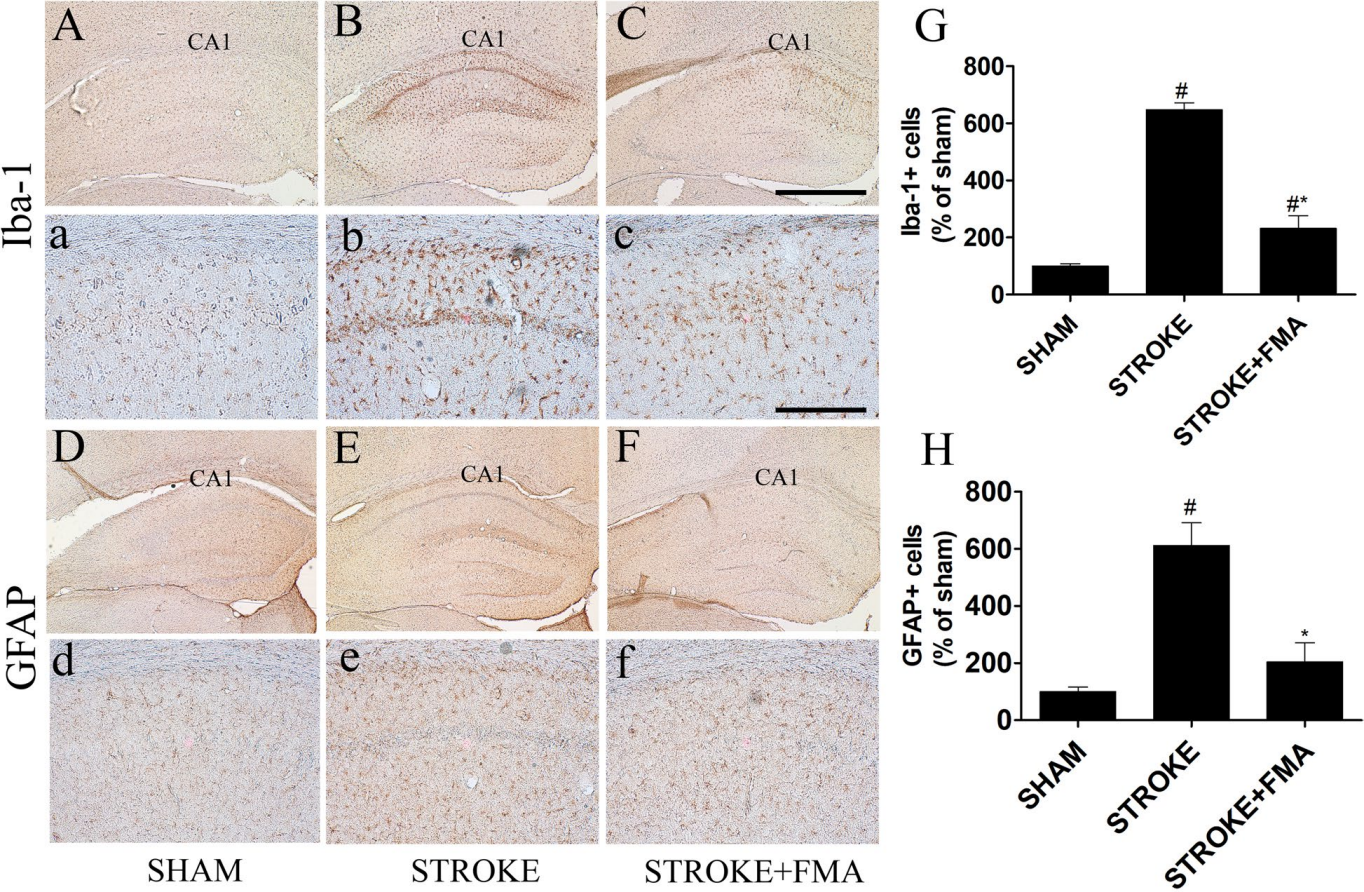
Effects of FMA on Iba-1 + microglia and GFAP + astrocytes in the CA1 area. Iba-1 + microglia in (**A**) sham group 10x (**a**) 200x, (**B**) TI-induced group 10x, (**b**) 200x, (**C**) TI-induced + FMA (400 mg/kg) group 10x (**c**) 200 × and GFAP + astrocyte in the CA1 area in (**D**) sham group 10x (**d**) 200x, (**E**) TI-induced group 10x, (**e**) 200x, (**F**) TI-induced + FMA (400 mg/kg) group 10x (**f**) 200x. In the TI-induced group, Iba-1 + were significantly increased and GFAP + cells showed larger and thicker processes in the CA1 region of the hippocampus compared with sham and FMA-pretreated groups. (**G)** Graph represent the immunoreactive Iba1 + and (**H**) GFAP + cells number (% of sham). Data are presented as mean ± SEM, *n* = 3/group, one-way ANOVA with a Tukey post-hoc tests; (#*p* < .05) compared with sham and (*p < .05) compared with Stroke

GFAP + and Iba-1 + neuronal cell arrangement was investigated in the hippocampus of all experimental groups (Fig. 3) to detect the activation of astrocytes and microglia. Inactivated astrocytes and microglia were found in all regions of the hippocampus in the sham group and easily detected by the absence of GFAP + (Fig. 3D,d) and Iba-1 + (Fig. 3A,a) cells. In the TI-induced group, significantly increased immunoreactive and hypertrophied GFAP + (Fig. 3E,e) and Iba-1 + (Fig. 3B,b) cells were observed in the hippocampus. Pretreatment with FMA extract for 7 days markedly decreased the number of hypertrophied immunoreactive astrocytes (Fig. 3F,f) and microglia (Fig. 3C,c) 5 days after TI induction in the CA1 area of the hippocampus and the shape and structure were unchanged compared with the TI-induced group.

### Effects of FMA on the viability of SH-SY5Y cells

**Fig. 4:**
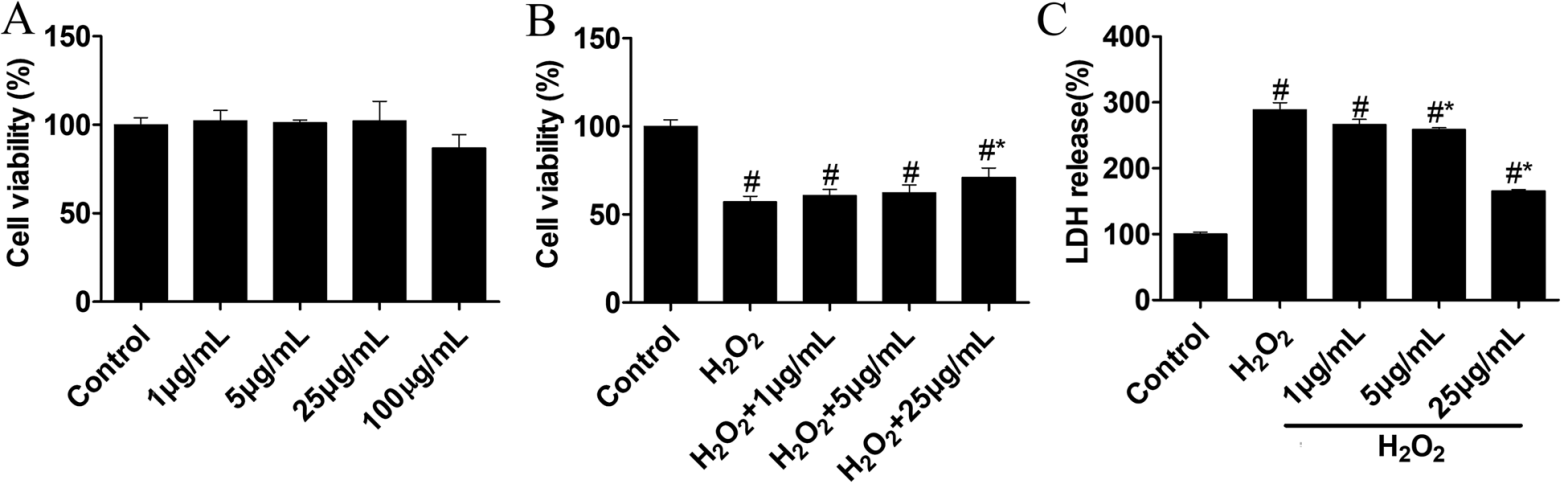
Effects of FMA on cell viability based on MTT and LDH assays in SH-SY5Y cells. **A** The toxic dose of FMA was estimated based on the MTT assay (% of control). **B** Cell viability against H_2_O_2_ toxicity was also evaluated using the MTT assay (% of control). **C** LDH secretion (% of control) as an oxidative stress marker was detected using LDH releasing assay. Data are presented as mean ± SEM, one-way ANOVA with a Tukey post-hoc tests. The experiment was repeated 3 times; (#*p* < .05) compared with control and (*p < .05) compared with H_2_O_2_

The MTT assay was used to evaluate the neuroprotective effects of FMA extract on H_2_O_2_‐mediated cytotoxicity in SH-SY5Y cells. The SH-SY5Y cells were treated with various FMA concentrations (1, 5, 25, or 100 μg/mL) for 24 h to examine the toxic dose and cell viability. A high FMA concentration (100 μg/mL) exhibited cytotoxic effects compared with untreated cells and lower concentrations (Fig. 4A). However, H_2_O_2_ (300 μM/mL)-mediated SH-SY5Y cell death was prevented by 2-h pretreatment with FMA (1, 5, or 25 μg/mL) in a dose-dependent manner (Fig. 4B). Thus, results indicated that FMA did not interfere with the viability of SH-SY5Y cells but protected against H_2_O_2_-induced toxicity.

### Anti-oxidative effects of FMA in SH-SY5Y cells

**Fig. 5:**
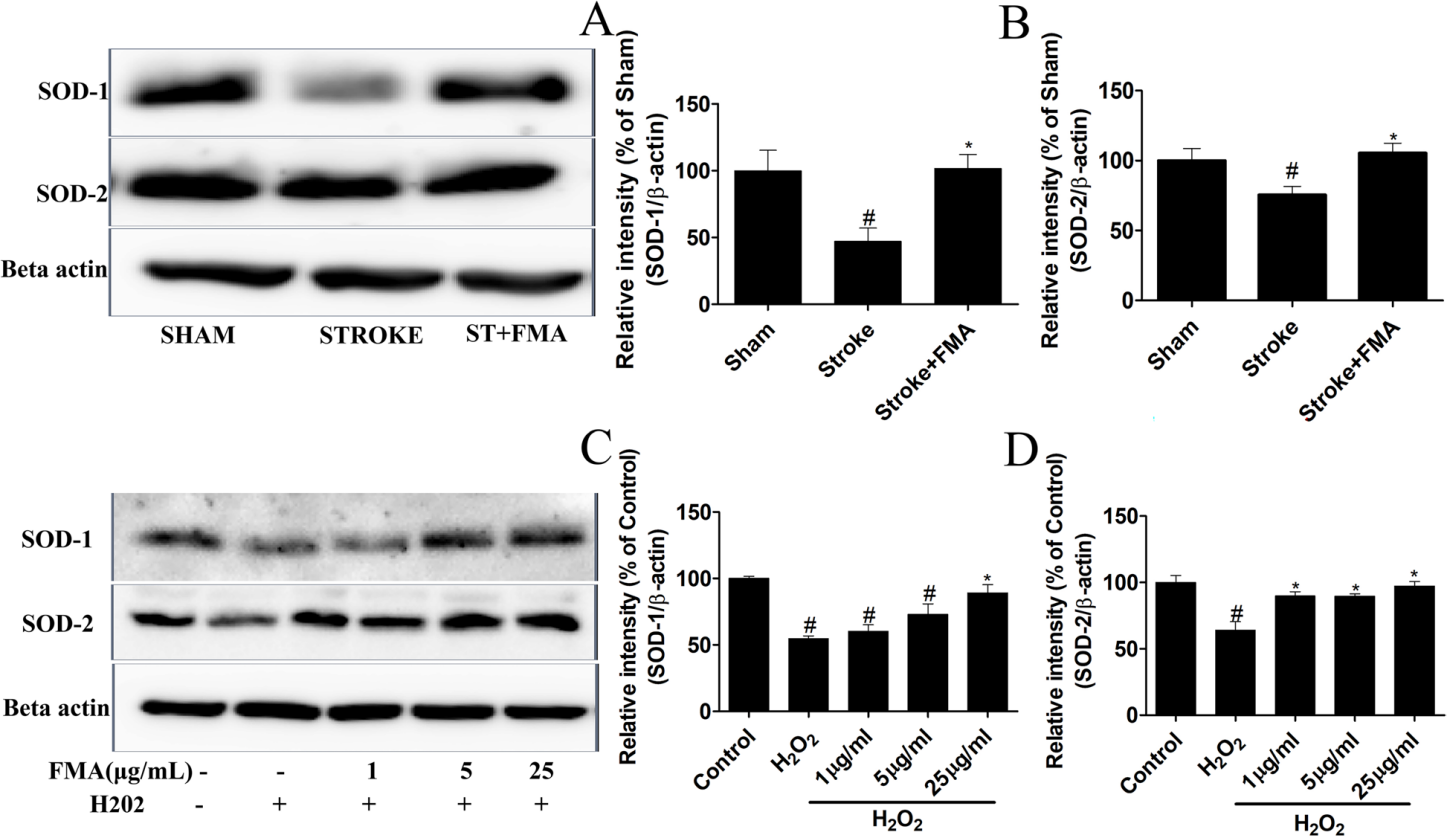
Effects of FMA on expression of antioxidant enzymes SOD-1 and SOD-2 in TI-induced hippocampus and H_2_O_2_-exposed SH-SY5Y cells. The relative intensity of (% of Sham) (**A**) SOD-1 and (**B**) SOD-2 proteins in the hippocampus and the relative intensity of (% of control) (**C**) SOD-1 and (**D**) SOD-2 in SH-SY5Y cells were increased after pretreatment with FMA extract. Data are presented as mean ± SEM, one-way ANOVA with a Tukey post-hoc tests. The experiment was repeated 3 times; (#*p* < .05) compared with control and (*p < .05) compared with H_2_O_2_

The effects of FMA on H_2_O_2_-mediated cytotoxicity were evaluated based on intracellular LDH secretion. The results showed that H_2_O_2_ treatment significantly increased intracellular LDH secretion (*P* > 0.05); however, FMA regulated LDH secretion triggered by H_2_O_2_ in a concentration-dependent manner (*P* > 0.05; Fig. 4C). Furthermore, the expression level of antioxidant enzymes SOD-1 and SOD-2 were estimated using western blot analysis in SH-SY5Y cells and the gerbil hippocampus. TI and H_2_O_2_ promoted oxidative damage in the gerbils (Fig. 5A, B) and SH-SY5Y cells (Fig. 5C, D) that caused decreased SOD-1 and SOD-2 protein expression levels. In addition, the expression of the two proteins was significantly increased (*P* < 0.05) after pretreatment with FMA extract in the gerbils and SH-SY5Y cells. Thus, the findings indicated that FMA had antioxidant effects in TI-induced gerbil hippocampus and H_2_O_2_-mediated oxidative stress in SH-SY5Y cells.

### FMA blocks the MAPK cascade

**Fig. 6:**
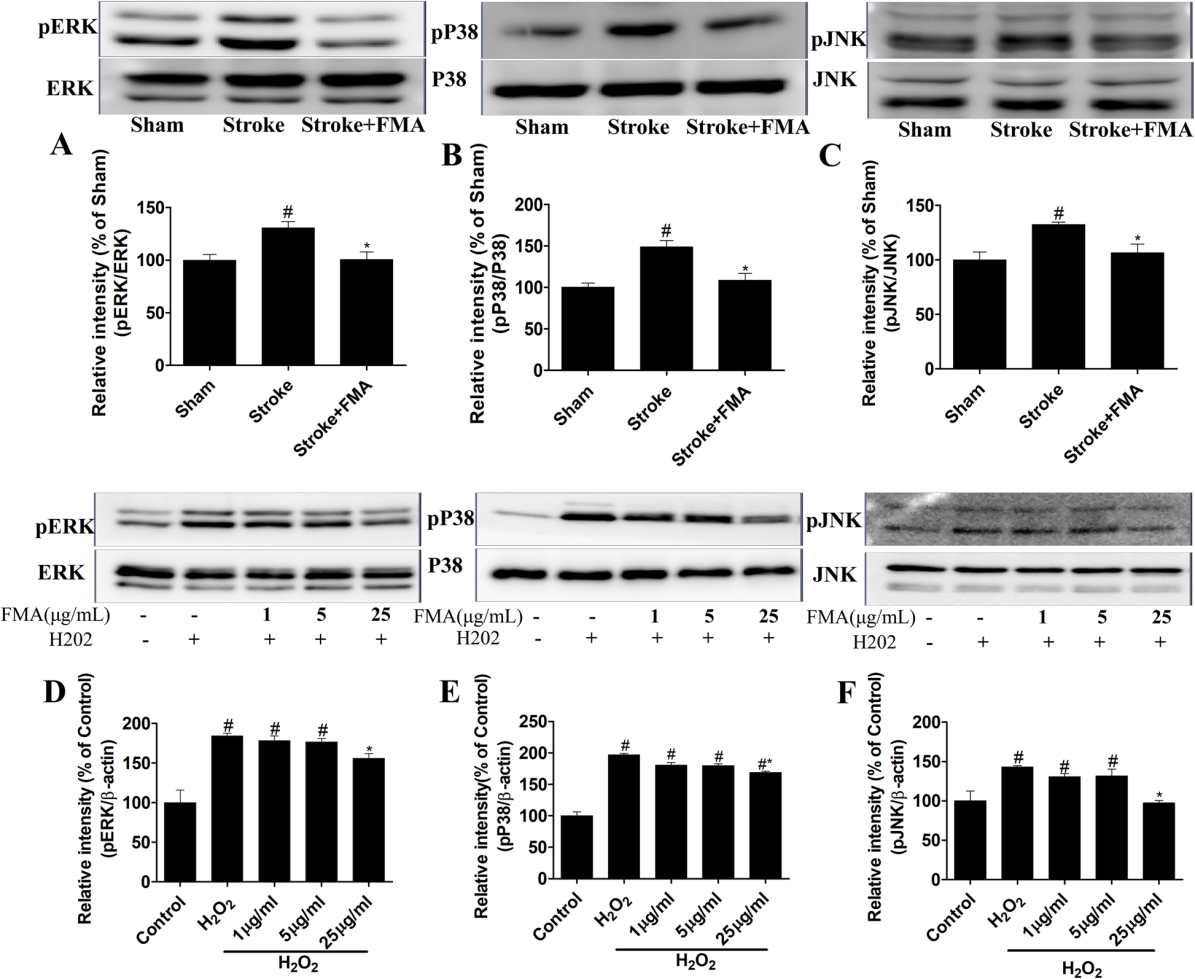
Effects of FMA on the expression of the phosphorylated form of ERK, JNK, and p38 proteins in TI-induced hippocampus and H_2_O_2_-exposed SH-SY5Y cells. The relative intensity of (% of Sham) (**A**) pJNK, (**B**) pP38, and (**C**) pERK proteins in the hippocampus and the relative intensity of (% of control) (**D**) pJNK, (**E**) pP38, and (**F**) pERK proteins in SH-SY5Y cells were decreased after pretreatment with FMA extract. Data are presented as mean ± SEM, one-way ANOVA with a Tukey post-hoc tests. The experiment was repeated 3 times; (#*p* < .05) compared with control and (*p < .05) compared with H_2_O_2_

H_2_O_2_-mediated ROS in SH-SY5Y cells can produce neurotoxicity by activating the MAPK cascade as shown in previous studies [24–26]. The results showed that phosphorylated proteins in the MAPK cascade (ERK1/2, JNK, P38) were significantly increased in the TI-induced gerbil’s hippocampus (Fig. 6 A, B, C) and H_2_O_2_ treated SH-SY5Y cells (Fig. 6 D, E, F). However, phosphorylation of ERK, JNK, and P38 proteins was significantly decreased (*P* < 0.05) after pretreatment with FMA extract in the hippocampus of gerbil and SH-SY5Y cells in a dose-dependent manner.

### Anti-apoptotic effects of FMA in SH-SY5Y cells

**Fig. 7:**
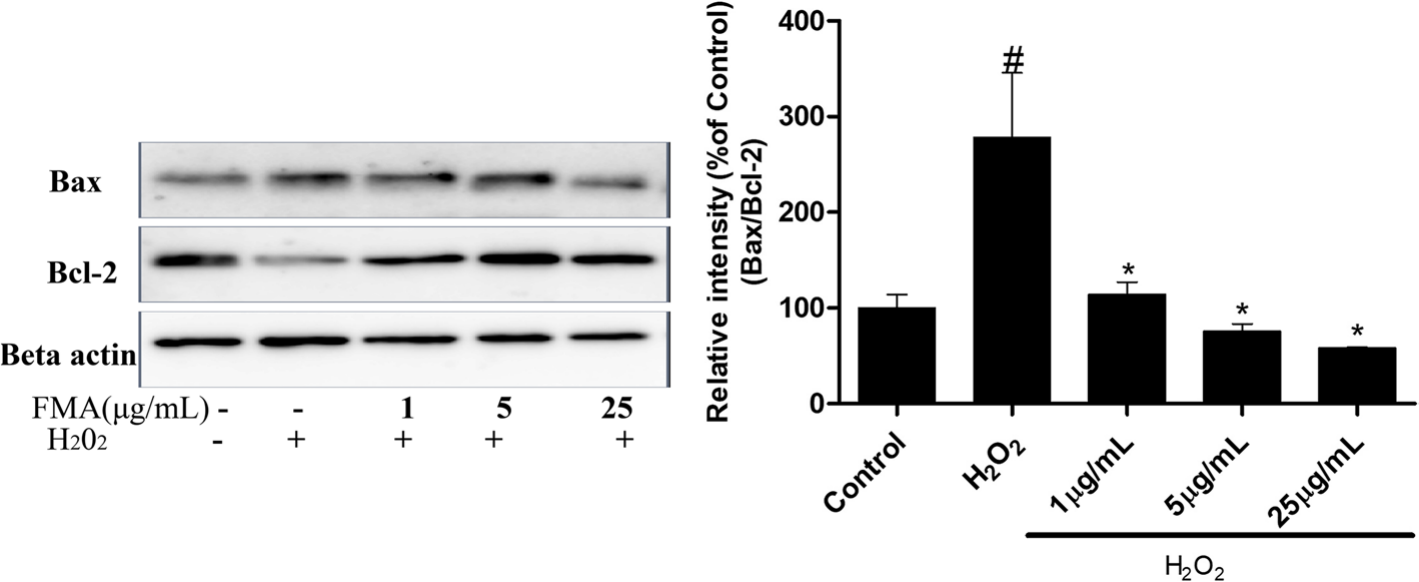
Effects of FMA on the expression of apoptosis proteins Bax and Bcl-2 in SH-SY5Y neuroblastoma cells. The relative intensity of (% of control) Bax and Bcl-2 proteins was decreased after pretreatment with FMA extract. Data are presented as mean ± SEM, one-way ANOVA with a Tukey post-hoc tests. The experiment was repeated 3 times; (#*p* < .05) compared with control and (*p < .05) compared with H_2_O_2_

The expression of apoptosis regulator protein Bcl-2 and apoptosis stimulator protein Bax was investigated using western blot analysis to determine the anti-apoptotic function of FMA. A low Bcl-2 and a high Bax protein expression was detected in H_2_O_2_-mediated SH-SY5Y cells. Furthermore, FMA extract significantly (*P* < 0.05) decreased the Bax/Bcl-2 ratio in a dose-dependent manner. Therefore, the results showed FMA regulated the Bax/Bcl-2 ratio (Fig. 7).

## Discussion

In the present study, the protective effects of FMA extract against TI-induced neuronal damage was investigated in a gerbil model of bilateral common carotid artery occlusion and H_2_O_2_-mediated neurotoxicity in SH-SY5Y cells. MA contains numerous enhanced natural phenolics and flavonoids, and due to the existence of different chemically active components, the entire plant has several therapeutic properties, including antioxidant, anti-inflammatory, gastroprotective, anti-fungal, and anti-bacterial effects [17–19]. In a previous study, fermentation of MA increased the active principle of rosmarinic acid, a water-soluble polyphenol that showed stress-induced strong antioxidative effects [19]. In the present study, the same FMA extract was used to investigate the neuroprotective role against TI.

TI in gerbils for 5–15 min induced neuronal cell death in the hippocampal CA1 area that was easily distinguished based on CV staining, immunohistochemistry, and F-J B histofluorescence [27–29]. In addition, F-J B is an effective marker for investigating neuronal degeneration in the brain following ischemic insults due to its strong affinity for degenerating neurons. Pretreatment with FMA extract markedly increased CV + cells and immunoreactive NeuN neurons and significantly decreased the number of F-J B + cells in the CA1 area of the hippocampus. These findings indicate that 5 min TI significantly increased neuronal cell death in the CA1 region and FMA pretreatment prevented the neuronal loss.

Numerous research results have confirmed that brain ischemia mediates excessive activation of glial cells [30–32] due to neuroinflammatory responses via secretion of different inflammatory mediators caused by ischemic insults [33, 34]. In this study, the activated number of astrocytes and microglia FMA-pretreated groups were significantly decreased in the CA1 area. In previous studies, the same pattern of glial cell activation after TI induction was observed [28, 35]. These results indicated that FMA extract has neuroprotective effects against TI insults that reduce gliosis.

The human neuroblastoma (SH-SY5Y) cell death model mediated by H_2_O_2_ is widely used to study neuronal cell death caused by oxidative stress and neuroprotective effects of natural products [36, 37]. The present study results indicated the H_2_O_2_-induced SH-SY5Y cell death was significantly reduced by pretreatment with FMA. In a previous study, FMA extracts showed protective effects against LPS-induced inflammation in RAW 264.7 cells [19]. LDH is an enzyme that exists in the cytosol and leaves the cell when the cell membrane is damaged by different types of stimuli [38]. In the present study, FMA pretreatment prevented H_2_O_2_-mediated cell membrane lysis and ultimately controlled LDH secretion. H_2_O_2_ plays a key role in the generation of ROS-associated oxidative stress. H_2_O_2_ enters cells rapidly and produces extremely reactive hydroxyl radicals, causing damage to intracellular components such as lipids, proteins, and DNA [39, 40]. In contrast, SODs are enzymes that can strongly scavenge superoxide radicals (O_2_^−^) and convert them to molecular oxygen and H_2_O_2_ [41]. In the present study, the intensity of SOD-1 and SOD-2 protein expression was significantly increased in the hippocampus and SH-SY5Y cells by pretreatment with FMA extract. In previous studies, reduced ROS generation and protein expression of the antioxidant enzymes SOD-1 and SOD-2 were shown to exert neuroprotective effects [9, 42]. Therefore, increased expression and activity of SOD-1 and SOD-2 might be associated with ROS reduction.

The MAPK cascade signaling pathway can be activated during inflammation induced by cell and tissue injury [43]. In particular, oxidative stress caused by ROS generation plays a key role in activating the MAPK pathway which can be estimated by the phosphorylated ERK, JNK, and p38 kinase levels [44]. ERK plays a major role in regulating cell apoptosis by releasing cytochrome c and/or caspase-8 activation [13]. In addition, JNK and p38 kinases are strongly associated with the apoptotic response caused by stress signals and DNA damage [45]. In previous studies, H_2_O_2_ was shown to activate the MAPK cascade in SH-SY5Y cells [46] and FMA extract and its active chemical substances downregulated MAPK protein expression levels [19]. In the present study, pretreatment with FMA significantly reduced increased phosphorylated ERK, JNK, and p38 MAPK levels in the hippocampus and SH-SY5Y cells, which supports the results of previous studies [19, 47]. Taken together, FMA pretreatment could reduce TI and H_2_O_2_-induced neurotoxic effects in gerbil hippocampus and SH-SY5Y cells respectively by inhibiting the MAPK signaling pathway. Bcl-2 family proteins, such as anti-apoptotic protein Bcl-2, and pro-apoptotic Bax, are actively involved in the mitochondria-related apoptosis process [48]. The permeability of the mitochondrial outer membrane and release of apoptotic agents from the mitochondrial interstitial space is regulated by Bax and Bcl-2 proteins which control apoptosis [49]. In the present study, Bcl-2 protein expression was increased and Bax protein expression decreased after pretreatment with FMA in H_2_O_2_-induced apoptosis of SH-SY5Y cells. These results indicate that Bax- and Bcl-2-dependent apoptotic pathways can be regulated by FMA extract.

## Conclusion

The results of the present study provide evidence that FMA inhibits neuronal cell death and provides almost normal hippocampal integrity against transient global ischemia and protects against H_2_O_2_-mediated neurotoxicity in SH-SY5Y cells. FMA pretreatment increased activation of antioxidant enzymes, regulated the expression of apoptosis-related proteins, and suppressed phosphorylation of MAPK proteins. These results indicate FMA could be a promising herbal medicine and potentially used clinically against ischemic stroke.

## Supplementary Information

**Additional file 1: Supplementary Figure 1. **The Uncropped immune blot data of antioxidant enzymes SOD-1 and SOD-2 in TI-induced hippocampus (Figure 5). The red arrow indicates the location of the target bands. **Supplementary Figure 2. **The Uncropped immune blot data of antioxidant enzymes SOD-1 and SOD-2 in H2O2-exposed SH-SY5Y cells (Figure 5). The red arrow indicates the location of the target bands. Edges are not visible in some blots because other parts of those blots were used for another experiment. **Supplementary Figure 3. **The Uncropped immune blot data of ERK, JNK, and p38 proteins in TI-induced hippocampus (Figure 6). The red arrow indicates the location of the target bands. **Supplementary Figure 4. **The Uncropped immune blot data of ERK, JNK, and p38 proteins in H2O2-exposed SH-SY5Y cells (Figure 6). The red arrow indicates the location of the target bands. Edges are not visible in some blots because other parts of those blots were used for another experiment. **Supplementary Figure 5. **The Uncropped immune blot data of apoptosis proteins Bax and Bcl-2 in H2O2-exposed SH-SY5Y cells (Figure 7). The red arrow indicates the location of the target bands.

## Data Availability

All data generated or analyzed during this study are included in this published article and its supplementary information files.

## Abbreviations

BAX: BCL2 Associated X
Bcl-2: B-cell Lymphoma 2
BSA: Bovine Serum Albumin
CA: Cornu Ammonis
CNS: Central Nervous System
CV: Cresyl Violet
DMSO: Dimethylsulfoxide
DNA: Deoxyribo Nucleic Acid
DPX: Dibutyl Phthalate Polystyrene Xylene
DW: Distilled Water
EMEM: Eagle’s Minimum Essential Medium
ERK: Extracellular Regulated Kinase
F-J B: Fluro-Jade B
FMA: Fermented *Mentha Arvensis*
GFAP: Glial Fibrillary Acidic Protein
HPLC: High-Performance Liquid Chromatography
H_2_O_2_: Hydrogen Peroxide
Iba-1: Ionized Calcium-Binding Adaptor Molecule 1
IHC: Immunohistochemistry
JNKc-Jun: N-terminal Kinase
LDH: Lactate Dehydrogenase
MA: *Mentha Arvensis*
MAPK: Mitogen-Activated Protein Kinases
MTT: 3-(4,5-Dimethylthiazol-2-yl)-2,5-Diphenyltetrazolium Bromide)
NeuN: Neuronal Nuclear Protein
PFA: Paraformaldehyde
RNA: Ribonucleic Acid
ROS: Reactive Oxygen Species
SDS-PAGE: Sodium Dodecyl-Sulfate Polyacrylamide Gel Electrophoresis
SOD-1: Superoxide Dismutase-1
SOD-2: Superoxide Dismutase-2
TI: Transient Ischemia
