# Supplementary material for: Fermented Mentha arvensis administration provides neuroprotection against transient global cerebral ischemia in gerbils and SH-SY5Y cells via downregulation of the MAPK signaling pathway
Source: BMC Complement Med Ther. 2022 Jun 25;22:172. doi: 10.1186/s12906-022-03653-7 (PMC9233811; doi:10.1186/s12906-022-03653-7)
Supplement: Supplementary file 1 — Additional file 1: Supplementary Figure 1. The Uncropped immune blot data of antioxidant enzymes SOD-1 and SOD-2 in TI-induced hippocampus (Figure 5). The red arrow indicates the location of the target bands. Supplementary Figure 2. The Uncropped immune blot data of antioxidant enzymes SOD-1 and SOD-2 in H2O2-exposed SH-SY5Y cells (Figure 5). The red arrow indicates the location of the target bands. Edges are not visible in some blots because other parts of those blots were used for another experiment. Supplementary Figure 3. The Uncropped immune blot data of ERK, JNK, and p38 proteins in TI-induced hippocampus (Figure 6). The red arrow indicates the location of the target bands. Supplementary Figure 4. The Uncropped immune blot data of ERK, JNK, and p38 proteins in H2O2-exposed SH-SY5Y cells (Figure 6). The red arrow indicates the location of the target bands. Edges are not visible in some blots because other parts of those blots were used for another experiment. Supplementary Figure 5. The Uncropped immune blot data of apoptosis proteins Bax and Bcl-2 in H2O2-exposed SH-SY5Y cells (Figure 7). The red arrow indicates the location of the target bands. [file 12906_2022_3653_MOESM1_ESM.pptx]

## Slide 1
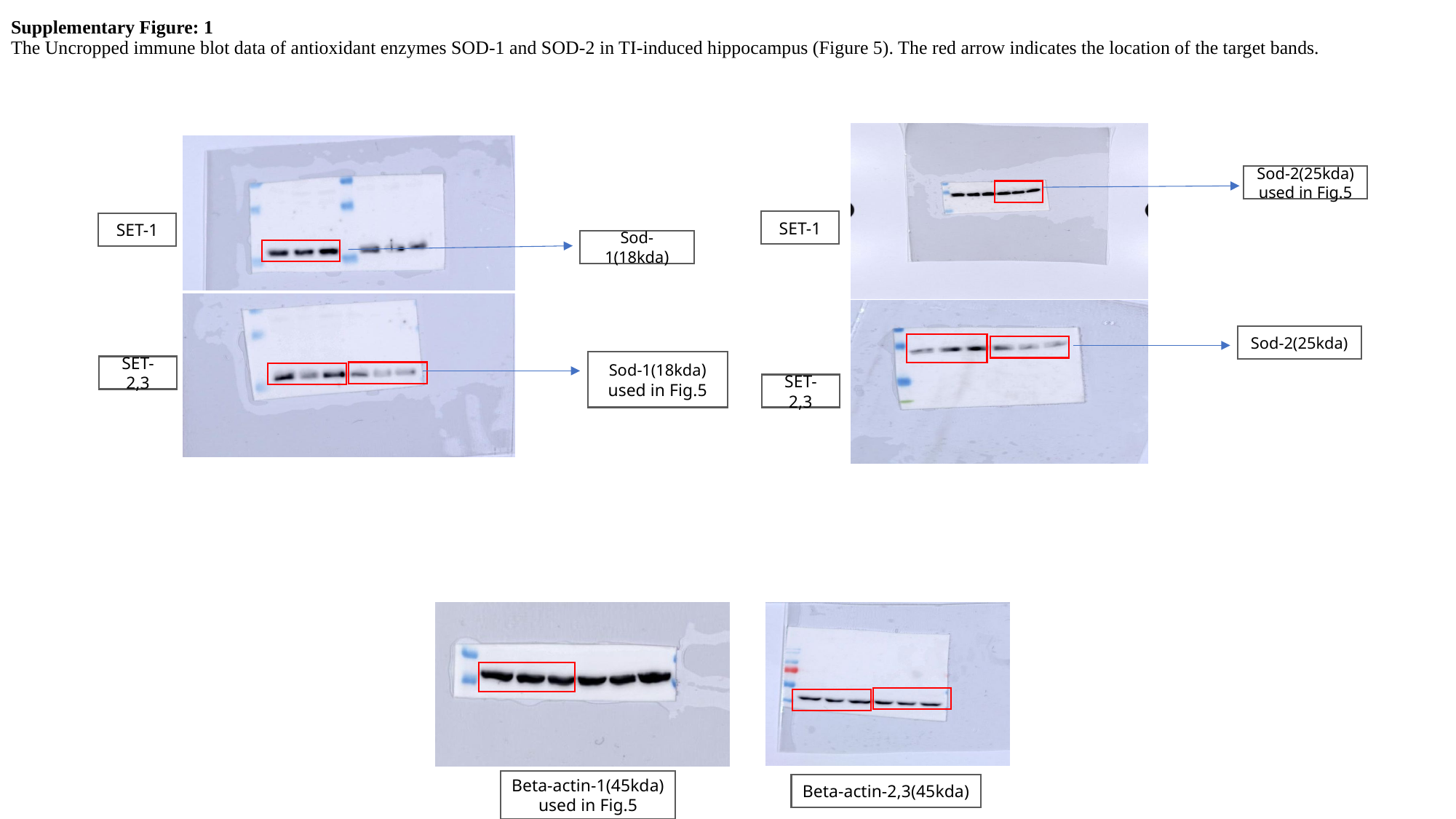

# Supplementary Figure: 1 The Uncropped immune blot data of antioxidant enzymes SOD-1 and SOD-2 in TI-induced hippocampus (Figure 5). The red arrow indicates the location of the target bands.
Sod-2(25kda) used in Fig.5
SET-1
SET-1
Sod-1(18kda)
Sod-2(25kda)
Sod-1(18kda) used in Fig.5
SET-2,3
SET-2,3
Beta-actin-1(45kda) used in Fig.5
Beta-actin-2,3(45kda)

## Slide 2
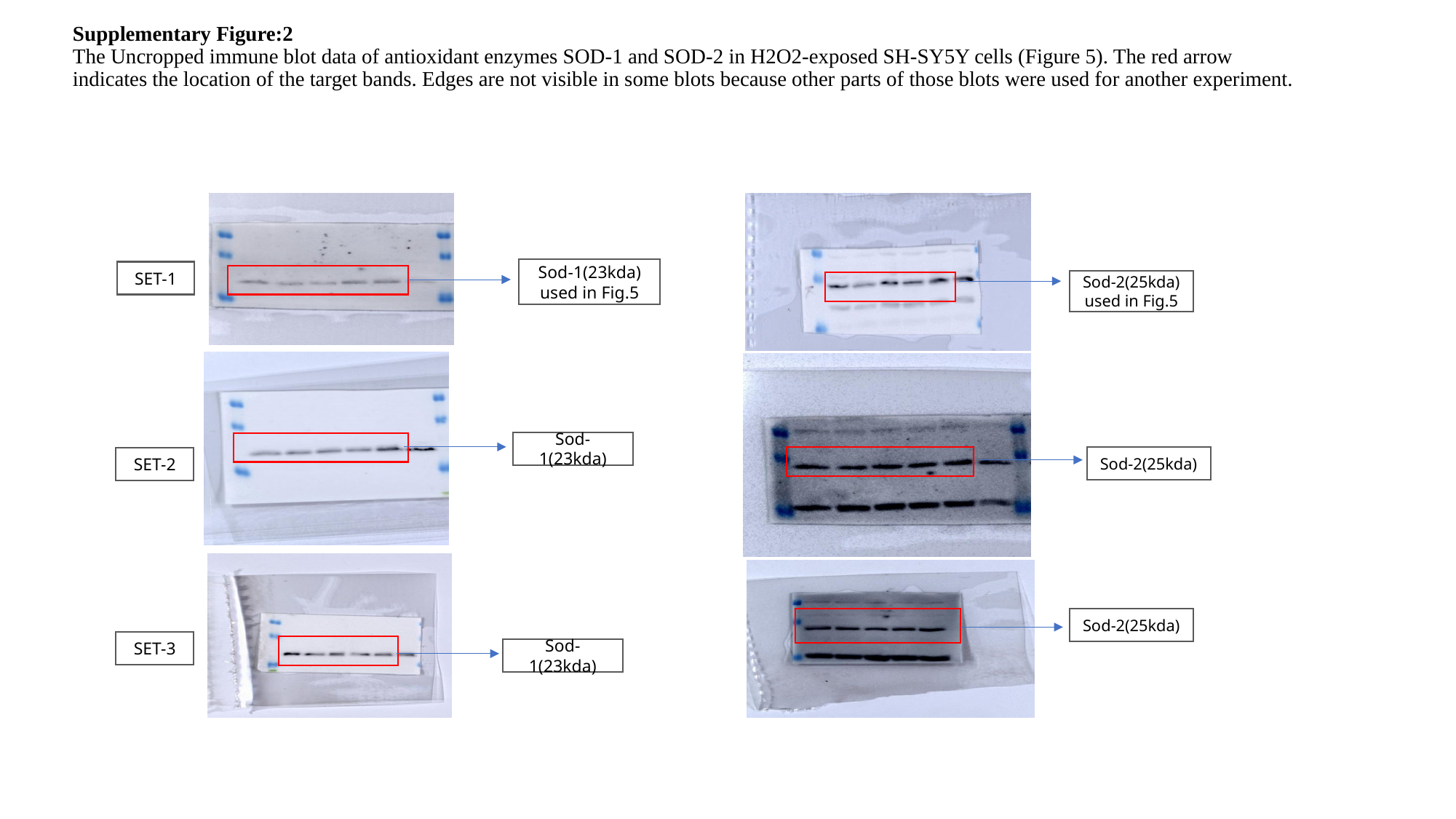

# Supplementary Figure:2 The Uncropped immune blot data of antioxidant enzymes SOD-1 and SOD-2 in H2O2-exposed SH-SY5Y cells (Figure 5). The red arrow indicates the location of the target bands. Edges are not visible in some blots because other parts of those blots were used for another experiment.
Sod-1(23kda) used in Fig.5
SET-1
Sod-2(25kda) used in Fig.5
Sod-1(23kda)
Sod-2(25kda)
SET-2
Sod-2(25kda)
SET-3
Sod-1(23kda)

## Slide 3
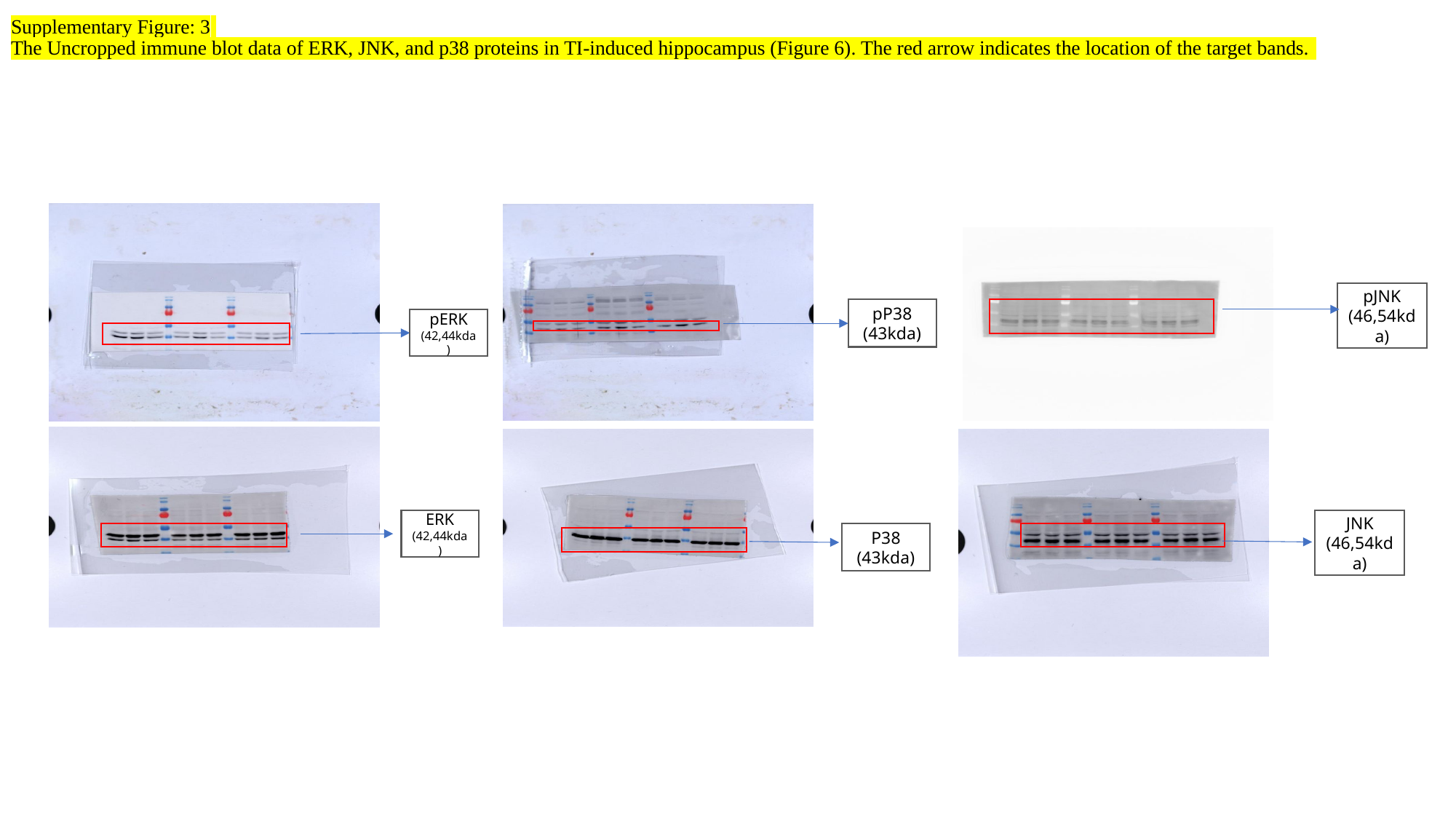

# Supplementary Figure: 3 The Uncropped immune blot data of ERK, JNK, and p38 proteins in TI-induced hippocampus (Figure 6). The red arrow indicates the location of the target bands.
pJNK
(46,54kda)
pP38 (43kda)
pERK (42,44kda)
ERK (42,44kda)
JNK
(46,54kda)
P38 (43kda)

## Slide 4
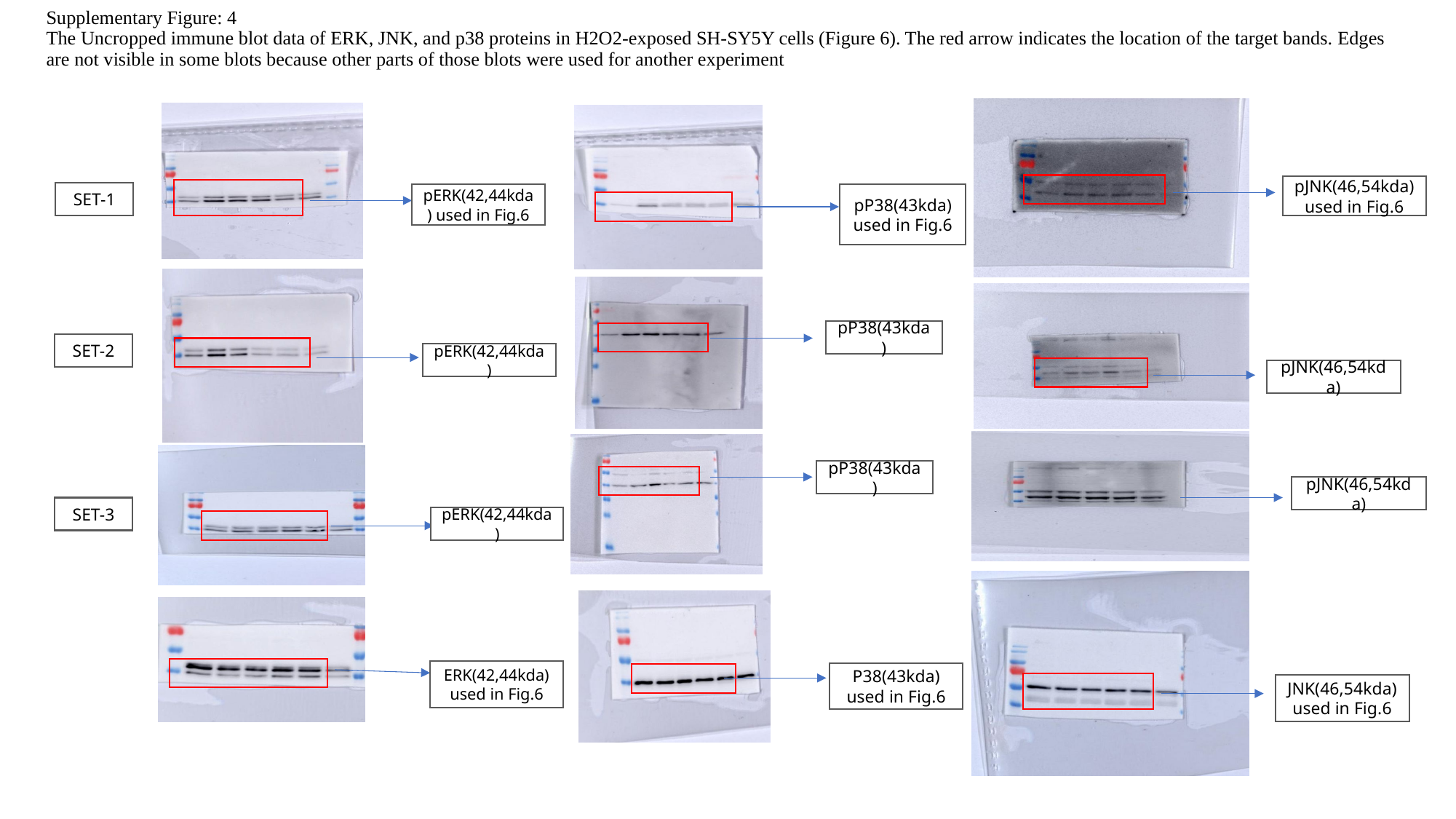

# Supplementary Figure: 4 The Uncropped immune blot data of ERK, JNK, and p38 proteins in H2O2-exposed SH-SY5Y cells (Figure 6). The red arrow indicates the location of the target bands. Edges are not visible in some blots because other parts of those blots were used for another experiment
pJNK(46,54kda) used in Fig.6
SET-1
pERK(42,44kda) used in Fig.6
pP38(43kda) used in Fig.6
pP38(43kda)
SET-2
pERK(42,44kda)
pJNK(46,54kda)
pP38(43kda)
pJNK(46,54kda)
SET-3
pERK(42,44kda)
ERK(42,44kda) used in Fig.6
P38(43kda) used in Fig.6
JNK(46,54kda) used in Fig.6

## Slide 5
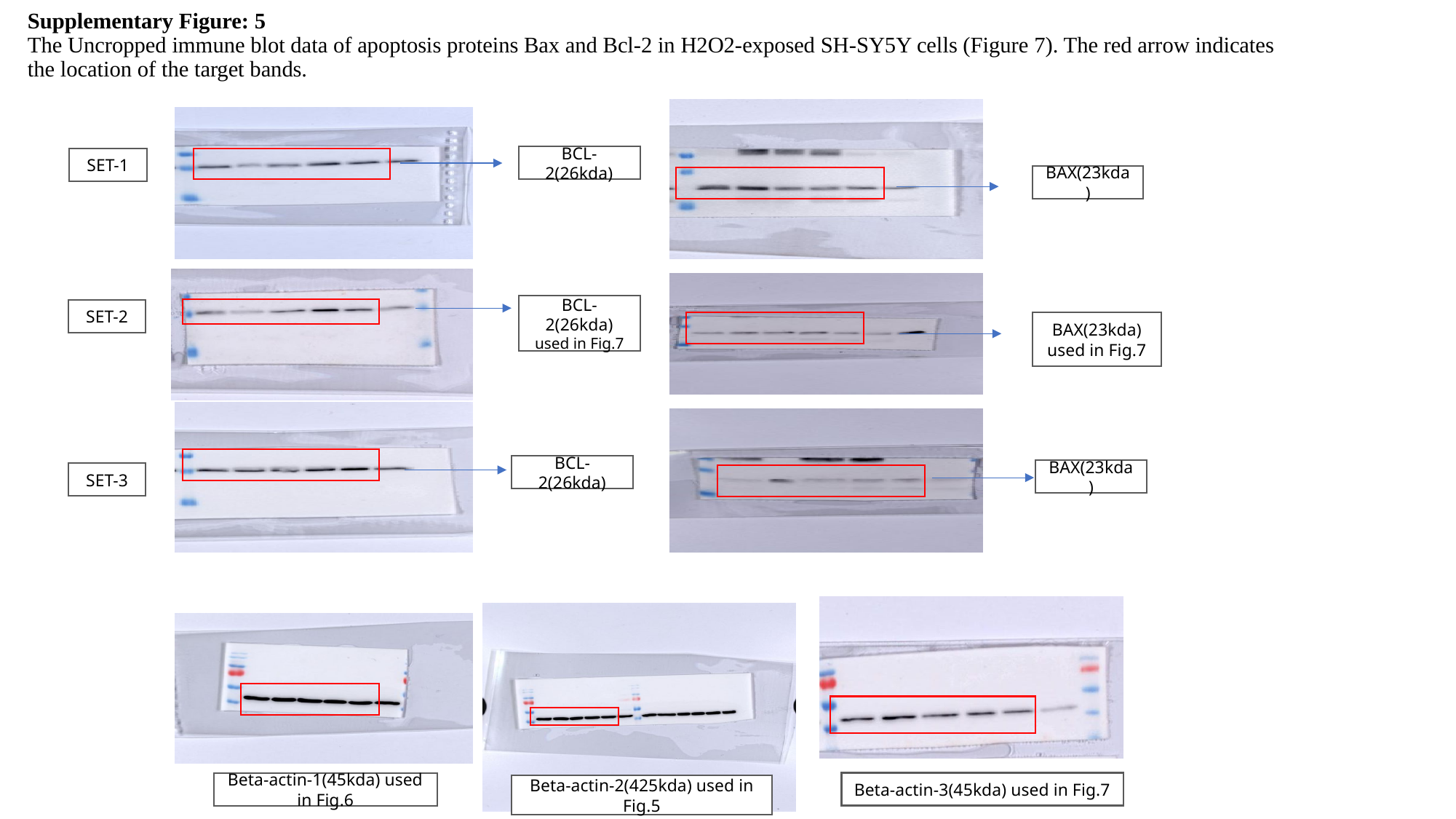

# Supplementary Figure: 5 The Uncropped immune blot data of apoptosis proteins Bax and Bcl-2 in H2O2-exposed SH-SY5Y cells (Figure 7). The red arrow indicates the location of the target bands.
BCL-2(26kda)
SET-1
BAX(23kda)
BCL-2(26kda) used in Fig.7
SET-2
BAX(23kda) used in Fig.7
BCL-2(26kda)
BAX(23kda)
SET-3
Beta-actin-3(45kda) used in Fig.7
Beta-actin-1(45kda) used in Fig.6
Beta-actin-2(425kda) used in Fig.5
